# DNA-methylation dynamics across short-term, exposure-containing CBT in patients with panic disorder

**DOI:** 10.1038/s41398-022-01802-7

**Published:** 2022-02-01

**Authors:** Sylvain Moser, Jade Martins, Darina Czamara, Jennifer Lange, Bertram Müller-Myhsok, Angelika Erhardt

**Affiliations:** 1 Department of Translational Research in Psychiatry, Max Planck Institute of Psychiatry, Munich, Germany; 2 International Max Planck Research School for Translational Psychiatry (IMPRS-TP), Munich, Germany; 3 Department of Health Data Science, University of Liverpool, Liverpool, UK; 4 Department of Psychiatry, Psychosomatics and Psychotherapy, Centre of Mental Health, Julius-Maximilians-University, Wuerzburg, Germany

**Keywords:** Epigenetics and behaviour, Clinical genetics

## Abstract

Interaction of genetic predispositions and environmental factors via epigenetic mechanisms have been hypothesized to play a central role in Panic Disorder (PD) aetiology and therapy. Cognitive Behavioral Therapy (CBT), including exposure interventions, belong to the most efficient treatments of PD although its biological mechanism of action remains unknown. For the first time, we explored the dynamics and magnitude of DNA-methylation and immune cell-type composition during CBT (*n* = 38) and the therapeutic exposure intervention (*n* = 21) to unravel their biological correlates and identify possible biomarkers of therapy success. We report transient regulation of the CD4 + T-Cells, Natural Killers cells, Granulocytes during exposure and a significant change in the proportions of CD4 + T cells, CD8 + T cells and B-Cells and Granulocytes during therapy. In an epigenome-wide association study we identified cg01586609 located in a CpG island and annotated to the serotonin receptor 3 A (HTR3A) to be differentially methylated during fear exposure and regulated at gene expression level with significant differences between remitters and non-remitters (*p* = 0.028). We moreover report cg01699630 annotated to ARG1 to undergo long lasting methylation changes during therapy (paired t test, genome-wide adj.*p* value = 0.02). This study reports the first data-driven biological candidates for epigenetically mediated effects of acute fear exposure and CBT in PD patients. Our results provide evidence of changes in the serotonin receptor 3 A methylation and expression during fear exposure associated with different long-term CBT trajectories and outcome, making it a possible candidate in the search of markers for therapy success. Finally, our results add to a growing body of evidence showing immune system changes associated with PD.

## Introduction

Panic disorder (PD) is a disabling psychiatric condition and characterized by repetitive and unpredictable panic attacks associated with psychological and somatic symptoms, such as fear of dying, feeling unreal, feel of being out of control, heart racing, shortness of breath sweating, feeling dizzy or trembling [1]. The majority of PD patients are affected by agoraphobia, where situations with limited escape or help possibilities are accompanied by intense fear, panic attacks, avoidance and anticipatory anxiety [2]. According to epidemiological studies, the lifetime prevalence ranges from 2.5 to 4% with women being affected twice as often as men [3, 4]. The appearance and course of panic symptomatology can be remitting-relapsing or chronic and comorbidity rates with other anxiety disorders and lifetime depression are high [5]. Age of onset for both PD and agoraphobia is in the adolescence and early adulthood impacting the individual personal and professional development at an early stage and causing high socioeconomic costs [6].

Anxiolytic antidepressants and cognitive behavioral therapy (CBT) including exposure interventions as treatment options show the best evidence for PD [7, 8]. However, the biological mechanisms underlying the effect of CBT and exposure interventions have not been elucidated yet and to date no biomarkers are available in the clinical routine to individually assign the best matching therapy to each patient or to supervise therapy-related progress.

Substantial evidence suggests a complex interplay between genetic and environmental factors in the etiology of PD and their role in the treatment-related outcomes [9]. The heritability is modest with 40–50% including frequent and rare variations of different effect sizes across the genome [10]. Thus, environmental influences are relevant for shaping biological processes which lead to PD pathology and recovery during therapy [11].

Epigenetics describes gene-regulatory mechanisms which are responsive to environmental influences, heritable, time-stable but also highly dynamic. This makes them interesting candidates to mediate effects of therapy in general and of exposure interventions in particular. Epigenetic changes include histone modifications, small/micro RNA-related gene silencing and DNA methylation (DNAm). The latter occurs at cytosines through addition of a methyl-group, mediated through DNA methyltransferases (DNMT). This process modulates gene expression by regulating accessibility of transcription factors to their binding sites [12]. DNAm in the promoter region often leads to reduced gene expression, whereas decreased or no methylation results in more active gene expression [13].

Few case-control epigenome-wide association studies (EWAS) are available for PD or PD-related symptomatology showing significant association of CpG sites in genes involved in cell-cycle regulation and immune system with overall small effect sizes [14–16]. A meta-analysis of two published EWAS [17] identified 61 significantly differentially methylated CpGs on epigenome-wide level. Several of those CpGs were annotated to genes which play a role in immune system regulations, such as the T-lymphokine- activated killer cell-originated protein kinase (*TOPK*) or in brain development and stress-induced depressive behavior, such as SMARCA5. The authors also observed differences in immune phenotypes and reported a decreased proportion of CD8 + T cells and an increased neutrophil-to-lymphocytes ratio in PD cases. While several other studies have reported similar results [18, 19], other disagreed [20], thereby highlighting a probably complex inter-play between PD and the immune system.

Despite these previous studies providing important evidence for DNAm and immune phenotypes regulation in PD, temporal dynamics of these changes during CBT and exposure still remain unclear. Indeed, to the best of our knowledge, only one longitudinal study assessed the evolution of DNAm over the course of CBT in PD [21]. While this study did not report statistically significant changes, it presented suggestive evidence for differential DNAm in *IL1R1* between treatment responders and non-responders. Identification and confirmation of such biomarkers could prove very useful to stratify and improve treatment in clinical practice. In summary, to date few data is available on the magnitude of the DNAm changes over the therapy and no studies are available on epigenetic changes during CBT interventions known to drive the therapeutic effects.

Consequently, the present study aims to investigate changes in DNAm and immune cell-types proportion acutely during the exposure intervention and longitudinally over the course of short standardized CBT. As a proof-of concept study with a relatively small sample size (*n* = 42), the changes in DNAm are investigated both at epigenome-wide level, aiming for uncovering new biological knowledge at the cost of a lower power, and at a more powerful but restricted candidate gene-approach.

## Methods

### Study participants

Fourty-two patients (males *n* = 15, females *n* = 27, mean age of 32 ± 10) (Supplementary Table 2) recruited in the Outpatient Clinic for Anxiety Disorders at the Max Planck Institute of Psychiatry between January 2014 and December 2015 were included in this study. As previously described in Martins et al. [22], all included individuals had a current primary DSM-IV-TR diagnosis of panic disorder (PD) with/without agoraphobia (PD/AG) and a clinical interview score >14 on the Hamilton Anxiety Scale (HAMA). Due to high comorbidity of the disorders, we use the term PD for the comorbid status throughout the text. Individuals with somatic disorders, pregnancy, personality disorders, current suicidal intent and other psychiatric disorders except other anxiety disorders or secondary mild or moderate depression were excluded from the study. Patients with anxiety disorders due to a medical or neurological condition or a comorbid Axis II disorder were also excluded. All patients included in the analysis were free of psychotropic medication and non-psychotropic drugs for at least 4 weeks before the therapy. Therefore, all medical conditions needing a pharmacological treatment as well as neurological conditions with putative damage of the central nervous system were exclusion criterion. The study was approved by the ethics committee of Ludwig-Maximilians-University in Munich, Project number 318/00. Written informed consent was obtained from all subjects.

### Treatment procedure and blood sample collection

Treatment followed a structured and empirically validated manual by Lang [23], which consists of 12 sessions of CBT (1–2 sessions per week, 6–8 weeks) as well as two booster sessions after 2 and 4 months. Therapy included two accompanied in vivo exposures (sessions 5 and 7) with therapists trained in exposure-based CBT. Exposure sessions were conducted outside the clinic, depending on the feared situation (e.g., subway, supermarket, height) and specific concern (e.g., fainting, asphyxiation, losing control). The type of exposure was determined by the patient, with the goal of highest fear provocation (for detailed information on therapy and exposure intervention see [24]).

**Fig. 1:**
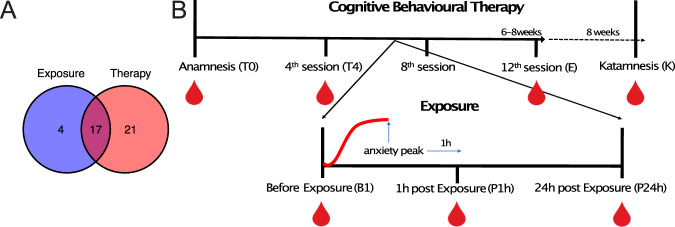
Cohort, experimental procedure and data collection. **A** Cohort**:** Our study cohort comprised 42 patients. 21 were included in the therapy part only, 17 in both the exposure and therapy and 4 only in the exposure part. **B** Experimental procedure: Treatment consisted of 12 sessions of CBT (1–2 sessions per week, 6–8 weeks) as well as two booster sessions after 2 and 4 months. Therapy included two accompanied in vivo exposures (sessions 5,7) conducted outside the clinic in a feared situation.Therapy course related blood samples were collected before the treatment (T0), after the forth session (T4), at the end of therapy (E) and 2 months after the therapy (K). Additionally, 3 blood samples were collected during the first exposure session (before the exposure basal (BE)), 1 h after the highest anxiety peak (post-exposure, P1h) and 24 h h after the exposure (P24h).

Therapy course related blood samples were collected before the treatment (T0), after the forth session (T4), at the end of therapy (E) and 2 months after the therapy (K) in 38 patients. Additionally, 3 blood samples were collected during the first exposure session (before the exposure (BE)), 1 h after the highest anxiety peak (post-exposure, P1h) and 24 h h after the exposure (P24h) (for detailed information see [22] and Fig. 1) in 21 patients. In total, 17 patients had their blood collected during both during the therapy and the exposure phase (Fig. 1). Blood collection, was performed at a fixed day time (9am) for every patient and every time point. Psychometric ratings of anxiety symptom severity were performed by a clinician not involved in the treatment using the German version of the clinician-rating Hamilton Anxiety Scale [25]. Patients were informed that their therapists would not have access to their responses on the measures. Therapy remission was reached if symptom ratings were below the cut-off 7. Remission was assessed after the end of therapy (12th session).

### DNA methylation data processing and quality control

Genomic DNA was extracted using the Gentra Puregene Blood Kit (Qiagen) and bisulfite converted with the Zymo EZ-96 DNA Methylation Kit (Zymo Research) to assess the methylations levels at ca. 480,000 CpGs sites with the llumina HumanMethylation450 BeadChip array, as described in [16]. Further processing and quality control, including removal of failing or cross-reactive probes, functional normalization, batch correction, beta-value calculation and cell composition estimation were performed as described in [16]. Beta-values were inverse-normal transformed to satisfy the normality assumption of linear models. A total of ca 425,000 CpGs remained after QC.

### Gene expression data processing and quality control

Blood RNA was collected at three timepoints (BE, P1h, P24h) and further processed as described in [26]. Blood RNA was hybridized to Illumina HumanHT‐12 v4 Expression BeadChips and Illumina’s GenomeStudio was used to export raw probe intensities. Further processing including normalization, batch correction and cell type composition estimation were performed as described in [22]. Expression values were inverse-normal transformed to satisfy the normality assumption of linear models.

### Changes in immune cell types composition during CBT

The white blood cell types composition was estimated from the DNAm data using the Houseman deconvolution method [27]. Linear mixed models (LMM) including sex, age, and daily cigarettes consumption as covariates, the respective cell-types proportions as outcome and first or second degree polynomial of the time as predictor were used to assess if cell types proportion changed during the exposure phase or therapy course. FDR-corrected *p* values derived from the best model, identified by likelihood ratio tests (LRT) and Akaike Information Criterion (AIC) as described in Supplementary Methods, are reported and used to evaluate regulation of cell-type proportions during the exposure or therapy.

For the therapy outcome, a LMM with the first order polynomial of time remission status and their interaction as predictor was fitted to the first (T0) and last (E) timepoint to test for differences between beginning and end of therapy stratified by remission status.

### Identification of differentially methylated CpGs during the course of exposure or therapy

Four LMMs including first- or second-degree polynomial of the time as predictor (see Supplementary Methods) were used to identify which CpGs were differentially methylated during the course of exposure or therapy and how they were regulated (i.e., in a linear or non-linear manner). These models were computed and compared using LRT and AIC as described in the Supplementary methods and included age, sex, number of cigarettes smoked per day immune cell types proportions and surrogate variables as covariates. The timepoints of therapy course and of the exposure phase were analyzed separately, as we hypothesized different types of regulation in these two time periods.

Paired-sample *t* tests comparing the first and last timepoints within the exposure or therapy phase were conducted to assess whether particular CpGs did return to their original levels after any kind of regulation. *P* values were FDR-corrected over the total number of test performed.

### Residualization of the methylation and gene expression data

Given the reported immune cell types changes in the course of exposure and therapy, we used linear regression models to regress them out from the DNAm and gene expression for visualization purposes and statistical models for which the inclusion of covariates is not possible (e.g., Paired-sample *t* tests).

### DMR analysis

We performed differentially methylated region (DMRs) analysis to group neighboring differentially methylated CpGs. Using the Comb-p software [28], we investigated regions containing at least 2 probes within a 750 base-pair window with a nominal *p* value for the best model smaller than 0.05. Because FDR-correction formally required by our model selection process would disturb the spatial correlation structure of the *p* values along the epigenome, which is needed in the Comb-p algorithm, we used uncorrected *p* values from the model in Eq. (2) (Supplementary Methods) for the exposure and Eq. (1) (Supplementary Methods) for the therapy as input for Comb-p. These models were selected as being the best model, as identified using LRT and AIC, for the majority of CpGs in that condition.

## Results

### White blood cell types regulation

To investigate whether exposure or CBT drive changes in the immune system state of PD patients, we assessed the evolution of different white blood cells proportions, derived from methylation data, over 3 time points in the exposure and 4 timepoints covering the whole therapy duration. We hypothesized that exposure and therapy are two distinct processes which differ regarding both their amplitude and duration, the exposure being an extreme but short event and the therapy being a steady but long-time process. We therefore analyzed them separately in this study.

**Fig. 2:**
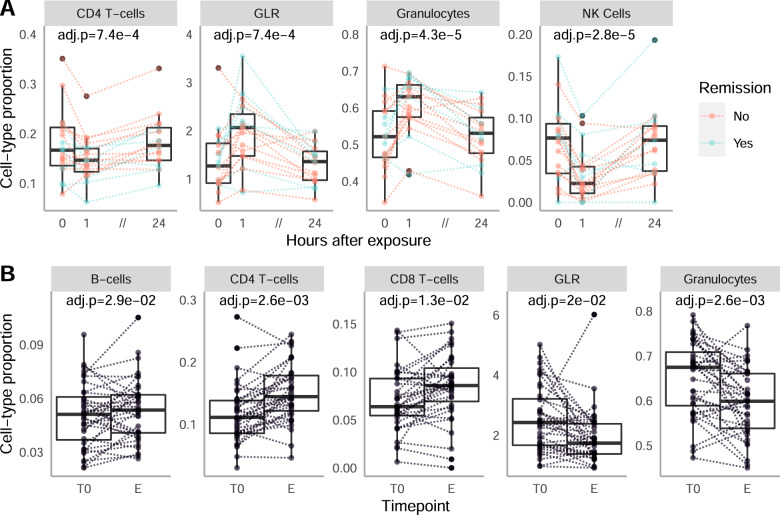
Changes in White blood cells during exposure and therapy. **A**. Regulation of CD4 + T-cells (left), Granulocytes (middle) and Natural Killer cells (right) during the exposure phase. Thicker lines represent the mean for the remitters (blue) and non-remitters (red). The adjusted *p* values are reported for the effect of Time in the selected LMM (see “Methods”). **B**. Regulation of CD4 + T-cells, CD8 + T-cells, B-cells, Granulocytes and of the granulocytes-to-lymphocyte ratio (GLR) during the course of therapy. Cell-types proportions were directly estimated from the methylation data. The adjusted *p* values are reported for the effect of Time in a LMM fitted on the first and last time point of the therapy.

We identified 3 immune cell types, the CD4 + T-Cells, the Natural Killers cells (NK) and the Granulocytes which were significantly regulated during the exposure (Table 1). Consequently, the granulocytes-to-lymphocyte ratio (GLR) was significantly regulated as well. The LMM with random intercept and second degree polynomial of the time as predictor (Eq. (2) in Supplementary Methods) was selected for these 3 cell types and the GLR, suggesting a non-linear regulation during the exposure, coherent with an acute event. Indeed, per individual cell-types proportions trajectories show a strong decrease (CD4T, NK cells, Fig. 2A) or increase (Granulocytes, GLR, Fig. 2A) one hour after the peak of anxiety before returning to pre-exposure value after 24 h. The magnitude of this effect, which happens over the course of one hour, is similar or greater than the changes observed in a time-scale of weeks during the therapy.

**Table 1:** Regulation of Immune Cell Type.

Cell type	*P* value time	Q value time	*P* value remission	Q value remission
**Exposure**				
CD8T	0.03	0.16	NA	NA
**CD4T**	**9.88** **×** **10**^**−5**^	**7.37** **×** **10**^**−4**^	NA	NA
**Natural Killer cells**	**1.00** **×** **10**^**−6**^	**2.80** **×** **10**^**−5**^	NA	NA
B-cells	0.40	0.73	NA	NA
Monocytes	0.76	0.81	NA	NA
**Granulocytes**	**3.09** **×** **10**^**−6**^	**4.32** **×** **10**^**−5**^	NA	NA
**GLR**	**1.00** **×** **10**^**−4**^	**7.38** **×** **10**^**−4**^	NA	NA
**Therapy**				
**CD8T**	**5.68** **×** **10**^**−3**^	**0.013**	0.53	0.68
**CD4T**	**7.54** **×** **10**^**−4**^	**2.64** **×** **10**^**−3**^	0.86	0.86
Natural Killer cells	0.45	0.45	0.32	0.68
B-cells	0.02	**0.03**	0.49	0.68
Monocytes	0.32	0.37	0.32	0.68
**Granulocytes**	**5.80** **×** **10**^**−4**^	**2.64** **×1** **0**^**−3**^	0.48	0.68
**GLR**	0.01	**0.02**	0.58	0.68

This table reports the results of the white blood cell types regulation analysis during the exposure and therapy respectively. The *P* value columns report the *p* value for the effect of time and of the remission status (for the therapy analysis) in the selected model The Q-value columns report the corresponding *p* value after multiple testing correction. LMMs were fitted on the 3 timepoints of the exposure and on the beginning and end of the therapy respectively. Statistically significant results are highlighted in bold.

For the course of the CBT, we report a significant change in cell-type proportion for Granulocytes, CD4T + and CD8 + T-cells, B-cells and for the GLR between the begin and the end of therapy (Table 1 and Fig. 2B). As expected for a gradual process such as therapy, the best selected model for all cell-types included the first-degree polynomial of the time as predictor (Eq. (1) in Supplementary Methods) and indicates a linear regulation. This is confirmed by the observation of the per-individual trajectories (Supplementary Fig. 1), which moreover show a higher inter-individual variance than that in the exposure, coherent with a weaker effect.

In the search of a biological marker for therapy outcome, we also investigated if individual differences in immune cell-types trajectories during exposure or therapy could be predictive of remission. However, no significant difference could be observed between remitters and non-remitters during the exposure (Fig. 2A red vs blue and Supplementary Fig. 1) or therapy course (Table 1, Supplementary Fig. 2).

### Identification of CpGs regulated during the course of exposure or therapy

With the aim of identifying possible biomarkers for therapy outcome as well as new mechanistical insights into the brain mechanisms triggered by exposure and therapy, we performed a methylome wide search for CpGs differentially methylated during exposure and therapy. We used LMMs for two reasons. Firstly, they allow to account for potential confounders, which was needed given the significant changes observed in the different white blood cell types proportions. Secondly, they allow to distinguish between linear and non-linear regulation, which might change the mechanistic interpretation of the findings.

No CpG was significantly regulated after multiple testing correction during the exposure or therapy in the LMMs analyses. Observations of the Quantile-Quantile diagnostics Plots (Supplementary Fig. 3), however, showed evidence of statistical signal and no signs of *p* value inflation. Based on this, we decided to rank the CpGs according to the strength of their statistical evidence. Similarly as described in [21], we combined this statistical ranking with a biological ranking based on the maximum absolute methylation difference between timepoints.

#### Exposure

**Fig. 3:**
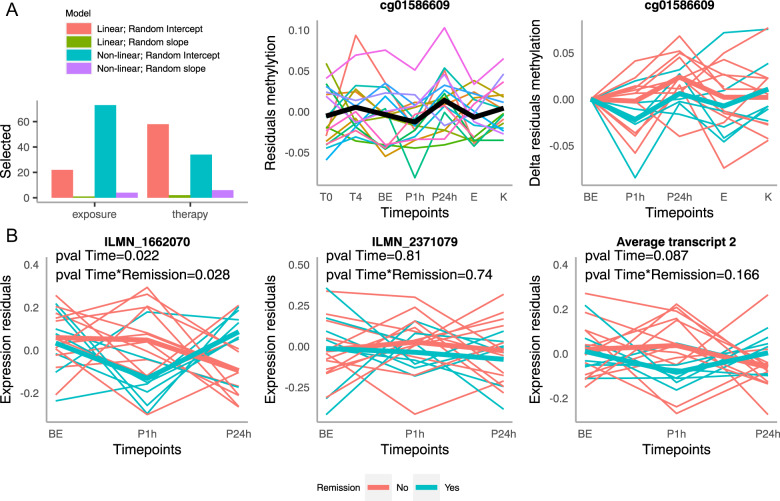
Regulation of HTR3A methylation and gene expression during the exposure phase. **A** Methylation: Left: Distribution of the selected models among the 100 best sum-ranked CpGs in the exposure and therapy phase. Middle: Evolution over time of cg cg01586609’s methylation residuals after regressing out the immune cell-type composition (referred as methylation residuals later) during the therapy (T0, T4, E, K) and exposure (BE, P1h, P24h) timepoints. The thick black line represents the mean over all samples. Right: Evolution over the exposure timepoints and subsequent therapy timepoints of the methylation residuals, centered at the value observed 1h before exposure. This graph represents the absolute changes in methylation during exposure, independent of the actual methylation levels. Thicker lines represent the mean for the remitters (blue) and non-remitters (red). **B** Gene Expression: Evolution over the therapy time points of the residuals of the gene expression data after regressing out the immune cell-types proportions of the ILMN_1662070 (left) and ILMN_2371079 (middle) probes annotated to the HTR3A gene and their average (right). Thicker lines represent the mean for the remitters (blue) and non-remitters (red).

We selected the top 100 CpGs (Table 2) in that sum ranking. These CpGs display both the highest statistical evidence for being regulated as well as potential for causal biological effects. The non-linear LMM was selected in more than 70% of this CpGs (Fig. 3A), which is coherent with the acute nature of the exposure and confirms that our approach is able to select regulated CpGs. Furthermore, we used paired sample t test on the residuals of the methylation (see methods) to test which of these 100 CpGs were regulated in a long-lasting manner (i.e. different between timepoint BE and P24h), as these CpGs are good candidates to participate in the effect of exposure on remission.

**Table 2:** Best-ranked CpGs for the exposure intervention and therapy.

CpG	*P* value	Q value	Bio rank	Stat rank	Sum rank	Gene
Exposure						
cg01586609	4.3 × 10^−5^	0.83	53	60	113	HTR3A
cg02279108	2.95 × 10^−5^	0.85	419	214	633	MNX1-AS1
cg27120833	1.63 × 10^−4^	0.89	35	620	655	ARFGAP3
cg13927247	2.2 × 10^−4^	0.87	448	290	738	DTWD2
cg03002526	1.9 × 10^−4^	0.87	673	305	978	HACE1
cg09855140	2.38 × 10^−4^	0.91	205	1044	1249	FKSG29
cg20611272	2 × 10^−4^	0.86	1068	251	1319	ODF1
cg07428959	9.7 × 10^−5^	0.90	445	885	1330	JADE1
cg13857354	5.7 × 10^−5^	0.91	172	1219	1391	RNPEPL1
cg13054007	2.4 × 10^−4^	0.87	1180	271	1451	SPRY2
Therapy						
cg01848660	1.00 × 10^−4^	1	124	42	166	C1D
cg23839680	3.03 × 10^−4^	1	180	153	333	PSPH
cg07205203	3.43 × 10^−4^	1	211	175	386	PPP1CB
cg03308839	2.34 × 10^−4^	1	584	107	691	NDE1
cg09160681	6.62 × 10^−4^	1	553	323	876	PARM1
cg12594803	1.74 × 10^−3^	1	28	873	901	DLG1
cg19676182	1.26 × 10^−3^	1	471	624	1095	CCDC149
cg20956594	8.78 × 10^−4^	1	701	425	1126	POMP
cg15210526	2.27 × 10^−4^	1	1054	103	1157	MPZL1
cg25853622	1.19 × 10^−3^	1	699	590	1289	LPP

This Table displays the 10 best CpGs ranked according to the cumulative, statistical and biological ranking (see “Methods”) for the exposure intervention and therapy respectively. The *p* value column reports the *p* value for the effect of time in the selected model. The Q value columns reports the corresponding *p* value after multiple testing correction. The Gene Column reports the gene annotated to the CpG.

The first CpG in both the overall and long-lasting CpGs ranking was annotated to the serotonin receptor 3 A gene, *HTR3A*. Methylation at cg01586609 showed a slow decrease between the 4th therapy time point towards the beginning of the exposure followed by an abrupt increase after the peak of anxiety to reach a summit 24 h thereafter (Fig. 3). We next investigated the DNAm dynamics of this CpG during exposure in remitters and non-remitters. When looking at the difference in DNAm compared to the first exposure time point, remitters show a stronger decrease of DNAm towards the peak of exposure, which is not fully recovered until the end of therapy. Given the low sample size and number of remitters (n_total_ = 21, n_remitters_ = 7), this trend is not statistically significant.

We next investigated if these DNAm changes had functional consequences at gene expression levels and looked at mRNA levels changes during exposure for 3 different probes annotated to the *HTR3A* gene. While the ILMN_1681492 probe associated with the first, longer transcript of the HTR3A gene did not show strong evidence of regulation (Supplementary Fig. 4), the ILMN_1662070 probe associated to the second transcript, coding for the canonical isoform of HTR3A, did. When looking at the residuals of gene expression after regressing out the immune cell-types proportions, expression drops drastically during the first hour of exposure only in remitters (Fig. 3B left). Afterwards, expression rises sharply in remitters towards the third time point while it decreases in non-remitters. Both, the regulation over-time and the difference in regulation between remitters and non-remitters, were statistically nominally significant (Linear mixed model; *p* value for Time^2^ = 0.022, *p* value for interaction of Time^2^ and remission status: 0.028).

The second probe annotated to the canonical isoform and located more distally at the very end of exon 9, did however not show the same evidence of regulation (Fig. 3B middle). Nevertheless, a clear trend is still observable when averaging the expression values of the two probes from the second transcript (Fig. 3C left) even if the regulation between timepoints and its interaction with the remission status are not significant anymore with this low sample size (*n* = 21 Linear mixed model; *p* value for Time^2^ = 0.087, *p* value for interaction of Time^2^ and remission status: 0.166).

#### Therapy

In contrary to the exposure, the first order LMM was selected for majority of the best 100 ranked CpGs for therapy which is coherent with the linear nature of the therapy process (Fig. 3A). Only 1 CpG was significantly differently methylated at the end of therapy as compared to the beginning in the paired-t test after multiple testing correction (cg01699630, ARG1; paired *t* test FDR corrected *p* value = 0.02). Additionally, the first and fourth CpGs in the sum ranking (Table 2) were annotated to the *C1D* and *NDE1* genes which have been respectively associated with Schizophrenia and epilepsy [29, 30]. Cg01848660, annotated to *C1D*, showed a linear increase during therapy in almost all patients but was also downregulated from the end of the exposure toward the end of therapy (Supplementary Fig. 6). Cg03308839, annotated to *NDE1*, showed an increase between the beginning (T0) and the fourth session of therapy (T4) followed by a decrease before the exposure and a stabilization in the subsequent timepoints (Supplementary Fig. 6).

### Candidate gene analysis

We also conducted a targeted analysis for CpGs associated with genes which expression we had found to be altered during exposure in a previous study [22] (Supplementary Table 1). No CpG withstood multiple testing correction. However, 2 CpGs annotated to the *MAL1D1* gene ranked among the top 10 best ranked CpGs for the therapy (Supplementary Table 2). Further analysis of the DNAm dynamics of these 2 CpGs over exposure and therapy timepoints showed a shared and highly consistent pattern across individuals (Supplementary Fig. 5), i.e. first decrease of DNAm during the beginning of therapy, followed by increase in anticipation of the fear exposure which continues during the exposure itself before a gradual decrease during the following therapy timepoints. This regulation is nominally significant for cg24577389 (LMM; *p* value fixed effect of time: 0.004, *p* value fixed effect of time2: 0.007269) and almost nominally significant for cg10418812 (LMM; *p* value fixed effect of time: 0.05, *p* value fixed effect of time^2^: 0.08).

### Differentially methylated regions analysis

It has been suggested that groups of spatially close co-regulated CpG probes, Differentially Methylated Regions (DMR), could be of particular biological relevance for several phenotypes. We therefore assessed if such clusters of co-regulated CpGs could be detected during therapy or exposure. Using the Comb-P software (Methods) we found six DMRs for exposure and one DMR for therapy (Supplementary Table 3). Of particular interest are the *SLC6A12* gene coding for a GABA transporter previously associated with negative symptoms in schizophrenia [31] and the insulin receptor substrate 2 which has been proposed to regulate dopamine cell morphology [32].

## Discussion

### Blood cell count

In the present study, we found a dynamic change of specific immune cell types during exposure and therapy. While some findings in regard to platelet reactivity indicators and red cell distribution width in PD compared to healthy controls suggest their diagnostic and predictive capacity [33, 34], investigations of immune markers in PD are still at the early stage. Petersen et al. analyzed immune cell proportions from two previous case-control DNAm studies in PD and observed significantly lower proportions of CD8 + T-lymphocytes, higher proportion of neutrophil as well as increased neutrophil-to-lymphocyte ratio in PD cases, however, after adjustment for age and sex, only CD8 + reduction was still significant [17]. The authors suggest that reduced CD8 + T cells are consistent with lower levels of IFN-gamma previously detected in a case-control study in PD [35]. Coherently, we report a significant regulation of the CD8 + T cells during therapy phase, with the proportion at the end of therapy being significantly higher than at the beginning. We also observe a significant decrease in Granulocytes and in the Granulocytes-to-lymphocytes ratio during therapy, in agreement with the trend described by Petersen and colleagues. As a whole, it seems that the immune cell-type composition changes over the course of therapy from a “PD-case-like” status towards a more “control-like” status.

In addition, we observe an increase of the CD4 + T cells proportion and decrease of the Granulocytes proportions during the therapy course. These changes are the opposite of the dynamics observed during the exposure phase. Indeed, CD4 + T cells shown an abrupt decrease one hour after the peak of anxiety, whereas the Granulocytes proportion increased dramatically. These variations in proportion are of greater magnitude than during therapy and happen in a very short time scale, pointing towards an acute response to the strong stress induced by exposure. Comfortingly, similar changes have been observed in response to acute stress in rodents [36, 37]. Taken together, our results suggest that exposure induces an acute stress response of the immune system whereas the therapy might lead to a gradual transition towards a “less-stressed” state of the immune system.

Interestingly, we did not observe any difference in any of the cell-types regulations between responders and non-responders. Whether this means that these regulations are physiological responses to stress, which are not malfunctioning in PD patients, or that they are part of the disease etiology but not affecting the therapy outcome, cannot be hypothesized from this study. Further studies, with higher sample size and inclusions of healthy control during the exposure phase are needed to clarify this question.

### Methylation regulation

In the present study, we conducted an epigenome-wide and a targeted candidate analysis of DNAm changes over the course of exposure and therapy. These two types of analysis have the potential to complement each other. The former being able to generate new hypotheses at the cost of a lower power, and the later allowing a higher power for a set of genes of interest.

We acknowledge that the sample size of our cohort of 42 patients provides only limited power for an epi- genome-wide analysis using LMMs. However, as this is the first study of this kind, and very little is known about therapy-related methylation in PD, we suggested that a hypothesis free approach could allow generating new hypotheses about possibly involved biological mechanisms and explore the magnitude of DNAm dynamics. Indeed, even if no CpGs reached epigenome-wide significance, the *p* values obtained from the LMMs are still indicative of the strength of the statistical evidence and allow to rank CpGs from the more to the less likely to be regulated. Combined with a ranking based on the magnitude of the observed changes, our statistical analysis allowed to uncover CpGs with a strong potential to be regulated. The validity of this approach is moreover confirmed by the very different distributions of selected LMMs for the best ranked CpGs during therapy versus exposure. Indeed, non-linear models were widely predominant in the exposure phase, consistent with an acute event, and linear models were predominant in the therapy phase, consistent with a long lasting process. This suggests that our models could indeed find meaningful biological changes.

We included several covariates, such as sex and age and smoking in our models, and used LMM which are able to account for unspecified variations among samples in order to correct for possible confounders in our study population. However, we cannot exclude that remaining confounders, such as diet, physical activity or stressful life events might have influenced our results. In particular if these variables were heterogeneously distributed between remitters and non-remitters. Moreover, the inclusion of healthy control group and randomized controlled experimental design with patients undergoing therapy and CBT free patients would be necessary to show that the DNAm and expression changes reported here are solely caused by the exposure or CBT. Nonetheless, we argue that the strict exclusion criteria of the study, such as the psychotrop-medication-free condition, the very homogenous study sample and very similar DNAm and expression trajectories observed across patients, plead in favor of a genuine effect of the interventions.

In the epigenome-wide association analysis of the exposure phase, the first CpG in both the overall and long-lasting CpGs ranking was annotated to the serotonin receptor 3 A gene (*HTR3A)* (product: 5HT_3_A receptor, 5HT3AR), which has been associated with numerous psychiatric disorders in human and related behavior in mice [38–42]. The 5-HT_3_ receptor is a ligand-gated ion channel composed of five subnits, in which 5-HT binding occurs to the extracellular N-terminus of the 5-HT_3A_ subunit [43]. 5HT3AR subtype is localized in limbic brain regions, such as the amygdala, hippocampus and throughout the cortex, closely involved in the regulation of panic states [44, 45]. The antagonism of the HT3 receptors display anxiolytic effects and blunted response to acute stress in rodents and primates, e.g., 5HT3AR null mice exhibit anxiolytic behavioral phenotype [46]. Furthermore, around 30% of GABAergic interneurons contain 5HT3AR and were suggested to influence cortical circuits during specific behavioral contexts [47]. A subset of these interneurons also co-expresses cholecystokinin (CCK) [48], a neuropeptide system used for panic induction via CCK4 and recently linked to metabolomic response during exposure [22, 49].

To investigate if these DNAm changes during exposure had functional consequences, we assessed the evolution of *HTR3A* gene expression in peripheral blood during exposure.

We report a strong decrease of *HTR3A* expression one hour after the peak of anxiety, compatible with a regulation through DNAm. Although both DNAm and gene expression changes are transient, with levels going back to initial values at the end of exposure, they could have long lasting implication during therapy and even predict therapy outcome. Indeed, transient changes in gene expression driven by DNAm changes have for example been showed to allow memory formation [50]. We therefore assessed whether *HTR3A* methylation and gene expression were differentially regulated in remitters vs non-remitters. And indeed, we observed a different dynamic of *HTR3A* expression with a nominally significant effect of the interaction between the remission indicator variable and the time in the LMM suggesting a different effect of the acute exposure on this serotonin receptor in remitters vs non-remitters. Decreased *HTR3AR* production in remitters suggest a pronounced anxiolytic effect in compare to non-remitted group after the exposure. From that point, antagonism of 5HT3AR might be a candidate mechanism to booster exposure effects by enhancing the anxiolysis and diminishing stress-induced deleterious effects in AD. In fact, there is evidence from pharmacological studies of improved antidepressive efficacy of Serotonine-Reuptake-Inhibitors, the first line treatment agents in most AD, after blockade of 5HT3AR [51]. Interestingly, the significant expression difference between remitters and non-remitters was found in a probe annotated to the canonical, functional, isoform of HTR3A, whereas the probe associated to the long isoform did not show significant regulation. This long isoform has been shown to be unable to form functional homomeric receptors but to be able to modify the response of heteromeric HTR3A receptors [52]. This suggest that the effect of HTR3A regulation on treatment outcome might be underpinned by a decrease of the canonical-homomeric form of HTR3A rather than by a modulation the serotonin response by heteromeric receptors.

In addition to *HTR3A*, a previous case-control study from our group identified methylation differences at CpGs annotated to two other serotonin receptors, namely *HTR1A* and *HTR2A* [16]. Taken together, these results suggest a shared involvement of the serotonin system in the etiology of PD and in response to acute fear during the exposure phase and further studies are needed to clarify a potential clinical application of 5HT3AR in therapeutic exposure and pharmacological treatment in AD.

Follow-up studies, with higher sample size would therefore be needed to replicate this preliminary finding and in vivo experiments would in addition be needed to assess whether these changes play a mechanistic role in therapy and exposure effects. In particular, the present study assessed the DNAm and gene expression in the blood as a proxy for brain DNA and expression, which are not readily accessible in humans. Several studies have shown that shown brain and blood transcriptomics to be very similar [53], and especially for receptors in general and for HTR2Ain particular [54], another members subtypes of serotonin receptors. Concordantly, the human protein atlas [55] reports similar HTR3A expression levels for the average of Peripheral Blood Mononuclear Cells (PBMC) and for several brain regions, including the cortex, thalamus and midbrain (https://www.proteinatlas.org/ENSG00000166736-HTR3A, accessed on 2021-10-20). However, it remains to be directly shown that the observed in the blood during the exposure and therapy phase are reflecting changes in the brain.

In the candidate analysis, we found two CpGs in the *MAD1L1* gene to be nominally regulated during the therapy. *MAD1L1* (mitotic arrest deficient 1like 1) dysfunction is associated with chromosomal instability and risk variations in this genes were linked to anxiety-related psychopathology [56], such as broad anxiety symptomatology [57] and neuroticism [58], but also further major psychiatric phenotypes, such as depression [59], bipolar disorder [60] and schizophrenia [61]. Furthermore, DNAm markers have been identified as associated with higher risk for PTSD in military male subjects in a longitudinal set up [62]. Snijders and colleagues reported decreased methylation at cg12169700, post trauma associated with decrease gene expression. On the contrary, we report decreasing methylation at cg10418812 and cg24577389 during the first phase of therapy followed by an increase during the exposure phase. These differences could be due to different effects of the initial trauma, therapy and exposure on the regulation of *MAD1L1* regulation. Nevertheless, these apparently opposite effect could participate in the same regulation process, given that cg12169700 is positioned inside the 16^th^ exon and cg10418812 is located respectively 5 kb before transcription start and in the 12^th^ exon. Indeed, whereas hyper-methylation of CpG in the promoter region is well understood and associated with gene expression suppression, the consequence of gene-body CpG methylation are still under study. Notably, several studies have shown gene-body CpG methylation to correlate with gene expression [63, 64]. Further studies, assessing gene expression during the therapy would be needed to conclude on the regulation of *MAD1L1* at the expression level during CBT.

In a complementary approach, we used paired t test to test for difference between the end and the beginning of the therapy only, ignoring the dynamics of the methylation in the intermediate time-points but looking for therapy-driven long-lasting changes. In the paired-t test, we obtained one CpG significantly differently methylated at the end of therapy as compared to the beginning in the gene *ARG1*. This gene is involved in the urea cycle and missense mutations in this gene cause serious developmental and neurological syndroms [65]. *ARG1* is important for macrophages specification and their effector functions [66] again highlighting that immunometabolism might be of importance in therapy-related effects.

## Conclusion

This is the first longitudinal study of DNAm and immune cell-type composition combining CBT course and acute fear exposure in PD patients. We firstly demonstrate that CBT and acute fear exposure do have measurable biological correlates, a critical argument in favor of their efficacy. Our results moreover provide evidence of the involvement of *HTR3A* in CBT success, calling for further experiment to dissect its mechanism of action and clarify potential clinical application. In addition, we also identified several genes with high potential of regulation during therapy to be investigated in further candidate studies. Finally, our study adds to the growing body of evidences linking PD and regulation of the immune system state.

## Supplementary information

Supplementary Material

Supplementary Table 1

## Data Availability

The code written to perform the analysis is available at https://github.com/sylvain-moser/CBT_DNAm.
