# Supplementary material for: DNA-methylation dynamics across short-term, exposure-containing CBT in patients with panic disorder
Source: Transl Psychiatry. 2022 Feb 1;12:46. doi: 10.1038/s41398-022-01802-7 (PMC8807826; doi:10.1038/s41398-022-01802-7)
Supplement: Supplementary file 1 — Supplementary Material [file 41398_2022_1802_MOESM1_ESM.docx]

# DNA-Methylation dynamics across short-term, exposure containing CBT in patients with panic disorder

Sylvain Moser^1,2^, Jade Martins^1^, Darina Czamara^1^, Jennifer Lange^1^, Bertram Müller-Myhsok^1,3^, Angelika Erhardt^1,4^

## Supplementary Methods:

### Linear Mixed Models for the identification of CpGs regulated during the course of exposure and therapy:

Linear Mixed Models (LMM) were used to identify which CpGs were regulated during the course of exposure or therapy and how they were regulated (i.e in a linear or non-linear manner). More precisely two different model with random intercept and respectively first, and second-degree polynomial for the time were fitted (Formula (1) and (2).

Cpg Methylation ~ Time + (1|NID) + cov_1_ + …+ cov_n_ (1)

Cpg Methylation ~ Time^2^ + (1|NID) + cov_1_ + …+ cov_n_ (2)

Third degree polynomial was not considered as it is almost impossible to fit with 4 time points. These two models were compared using the Likelihood Ratio Test (LRT), as they share the random effect structure. In addition, a third model with the first degree time polynomial but random effect for the individual on the intercept and the slope was fitted (Formula (3)).

Cpg Methylation ~ Time + (Time|NID)+ (1|NID) + cov_1_ + …+ cov_n_ (3)

Finally, a forth model with the second degree time polynomial fixed effect and random effect for the individual on the intercept and the slope was fitted (Formula (4)).

Cpg Methylation ~ Time^2^ + (Time^2^|NID)+ (1|NID) + cov_1_ + …+ cov_n_ (4)

The best of the two random intercept model (as selected by the LRT) was compared to the two random slope models using the Akaike Information Criterion (AIC), as these models do not share the same random structure. The model with the lowest AIC was selected as overall best model. Finally, multiple testing correction was performed for all CpGs across the 4 models using BH method for FDR correction. The CpGs were then ranked according the FDR-corrected p-value derived from the best model.

All models were fitted using the R package lme4 and included sex, age and the white blood cells proportion as covariates. Surrogate variables, estimated from the methylation data using the R package sva were also included as covariates to account for residual heterogeneity (Leek & Storey, 2007). The SVs and the immune cell-types were both included in the models as they account for different sources of heterogeneity (see Supplementary Figure 7).

The p-value for the beta coefficient of the fixed effect of time were computed using the LmerTest implementation of the F-test with the Satterwhaite’s estimation of the degrees of freedom.

### Linear Mixed Models for gene expression regulation during the course of exposure:

LMMs were used to assess the regulation of selected target genes at the expression level during the exposure. In this case, only one LMM including the second degree time polynomial fixed effect and random effect for the individual on the intercept and the slope was fitted (Formula (5)).

(5) Probe expression ~ Time^2^ + (Time^2^|NID)+ (1|NID) + cov_1_ + …+ cov_n_ (4)

This model included sex, age, the agoraphobia diagnosis and the remission status as covariates. The assumption of equal variance between groups, in this case between the remission status strata, was tested using F-test.

## Supplementary Figures:

**Supplementary Figure 1:** Changes in White blood cells during exposure and therapy: individual data.

**A.** Regulation of immune-cell types proportions during the exposure. Thicker lines represent the mean for the remitters (blue) and non-remitters. **B.** Regulation of immune-cell types proportions during the course of therapy. Thicker lines represent the mean for the remitters (blue) and non-remitters (red). Cell-types proportions were directly estimated from the methylation data.


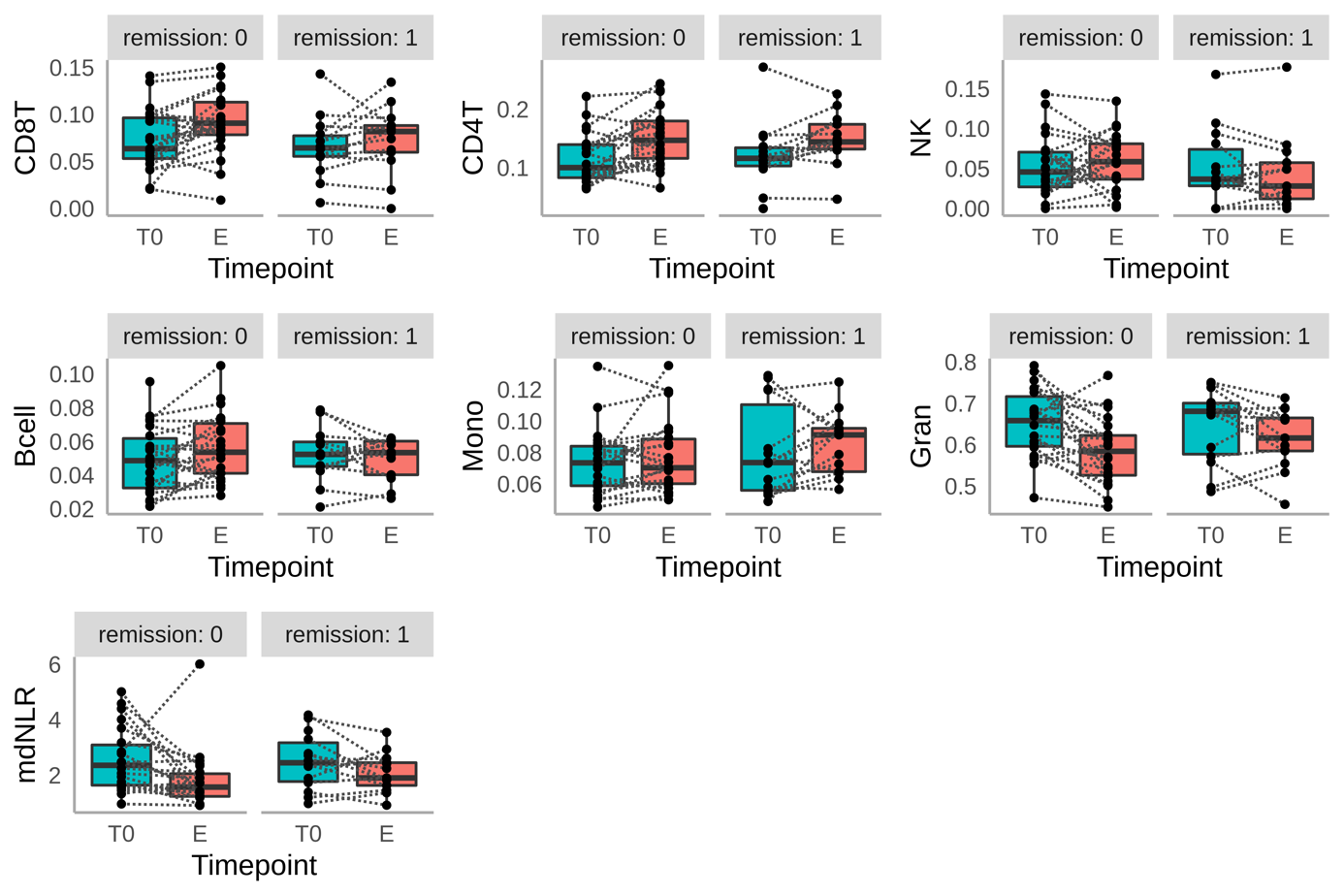


**Supplementary Figure 2:** Changes in White blood cells between beginning and end of the therapy stratified by therapy response:

Distribution of the respective immune-cell types proportions at the beginning (T0) and End (E) of the therapy for remitters (remission=1) and non-remitters (remission=0).

**Supplementaty Figure 3**: Quantile-Quantile diagnostics plots for the LMM used to identify regulated CpGs during exposure and therapy.

A. Exposure. Quantile-Quantile plots (QQ-plots) for the 4 LMMs used to identify regulated CpGs during the exposure. The X axis represent the expected p-values under the null hypothesis of no regulation. The X axis represent the observed p-values. B. Therapy. Quantile-Quantile plots (QQ-plots) for the 4 LMMs used to identify regulated CpGs during the therapy.

**Supplementary Figure 4:** **Regulation of HTR3A methylation and gene expression during the exposure phase**

Evolution over the three exposure timepoints of the ILMN_1681492 probe associated with the first transcript of the HTR3A gene. The immune-cell types proportions were regressed out of the expression using linear regression prior to plotting. Thicker lines represent the mean for the remitters (blue) and non-remitters (red).

Supplementary Figure 5: **Regulation of MAD1L1 methylation over therapy and exposure phase:**

Evolution over time of the methylation residuals after regressing out the immune cell-type composition at the cg10418812 (left) and cg24577389 (right) probes annotated to the MAD1L1 gene during the therapy (T0, T4, E, K) and exposure (BE, P1h, P24h) timepoints. The thick black line represents the mean over all samples.

**Supplementary Figure 6:** **Regulation of C1D and NDE1 methylation during the exposure and therapy.**

Evolution over time of the methylation residuals after regressing out the immune cell-type composition at the cg01848660 (annotated to the C1D gene) and cg03308839 (annotated to the NDE1 gene) during the therapy (T0, T4, E, K) and exposure (BE, P1h, P24h) timepoints. The thick black line represents the mean over all samples.

**Supplementary Figure 7: Proportion of SVs variance explained by immune cell-types**

In order to prevent over-correcting the LMMs by including the SVs and the immune cell-types as covariates, which could be very colinear, we investigated whether they were picking up different sources of heterogeneity. To do so, for 1000 randomly sampled CpGs we fitted linear models with the each of the SVs as dependent variable and the immune cell-types as predictor. We then looked at the adjusted R^2^ value, which can be interpreted as the percentage of the variance of the SV which is explained by the immune cell-types. In both the exposure (A) and the therapy(B) the R^2^ are low, indicating that the SVs and the immune cell proportions are representing different sources of heterogeneity and are not colinear. We therefore decided to include both as covariates in the LMMs.

## Supplementary Tables:

| Therapy |  | Non-remitters | Remitters | No remission status |
| --- | --- | --- | --- | --- |
|  | Participants | 24 | 14 | 0 |
|  | Age | 32.4 ± 10.3 | 34.7 ± 10.1 | 0 |
|  | Sex (F/M) | 18/6 | 7/7 | 0 |

| Exposure |  | Non-remitters | Remitters | No remission status |
| --- | --- | --- | --- | --- |
|  | Participants | 12 | 7 | 2 |
|  | Age | 32.8 ± 11 | 31.1 ± 6.6 | 26.5 ± 6.3 |
|  | Sex (F/M) | 10/2 | 4/3 | 1/1 |

Supplementary Table 2 **Demographics of study cohort:**

This table reports the demographic characteristics of the individuals included in the exposure and therapy analysis, stratified on their remission status.

| **CpG** | **P-value** | **Q-value** | **Gene** |
| --- | --- | --- | --- |
| cg06446466 | 2.15×10^–5^ | 0.31 | RFXANK |
| cg19029859 | 0.00030 | 1 | UBE3C |
| cg18571451 | 0.00111 | 1 | LDB2 |
| cg10418812 | 0.00148 | 1 | MAD1L1 |
| cg24577389 | 0.00173 | 1 | MAD1L1 |
| cg19870567 | 0.00175 | 1 | ATP6V0E1 |
| cg16419418 | 0.00181 | 1 | MGST1 |
| cg18645906 | 0.00266 | 1 | BST1 |
| cg25457331 | 0.00280 | 1 | GMPR |
| cg15636040 | 0.00301 | 1 | ACP6 |

Supplementary Table 3 **Targeted analysis for genes regulated at the expression level during therapy (Martins et al., 2019):**

This Table displays the 10 best CpGs in the therapy candidate gene analysis on gene shown to be regulated at the expression levels during exposure. The CpGs are ranked according to their nominal p-value. The p-value column reports the p-value for the effect of time in the selected model. The Q-value columns reports the corresponding p-value after multiple testing correction. The Gene Column reports the gene annotated to the CpG.

| Chromosome | Start | End | number of CpGs | Nearest Gene | Phase |
| --- | --- | --- | --- | --- | --- |
| 2 | 203130903 | 203130956 | 2 | NOP58 | exposure |
| 5 | 78985425 | 78985593 | 9 | CMYA5 | exposure |
| 12 | 322641 | 322889 | 6 | SLC6A12 | exposure |
| 12 | 56617867 | 56618155 | 9 | NABP2 | exposure |
| 13 | 110386152 | 110386268 | 3 | IRS2 | exposure |
| 20 | 33422404 | 33422628 | 2 | HMGB3P1 | exposure |
| 14 | 65569312 | 65569400 | 6 | MAX | therapy |

Supplementary Table 4 **Differentially Methylated Regions**:

DMR were identified as sequence of two or more nominally significantly regulated CpGs within a 750bp window and annotated to the nearest gene.
